# Metagenomic Next-Generation Sequencing for Diagnosing Infections in Lung Transplant Recipients: A Retrospective Study

**DOI:** 10.3389/ti.2022.10265

**Published:** 2022-02-10

**Authors:** Chun-Rong Ju, Qiao-Yan Lian, Wei-Jie Guan, Ao Chen, Jian-Heng Zhang, Xin Xu, Rong-Chang Chen, Shi-Yue Li, Jian-Xing He

**Affiliations:** ^1^ State Key Laboratory of Respiratory Disease, National Clinical Research Center for Respiratory Disease, Guangzhou Institute of Respiratory Health, The First Affiliated Hospital of Guangzhou Medical University, Guangzhou, China; ^2^ Department of Thoracic Surgery, Guangzhou Institute of Respiratory Disease, First Affiliated Hospital of Guangzhou Medical University, Guangzhou, China; ^3^ Department of Respiratory and Critical Care Medicine, First Affiliated Hospital of Southern University of Science and Technology, Second Clinical Medical College of Jinan University, Shenzhen People’s Hospital, Shenzhen Institute of Respiratory Diseases, Shenzhen, China

**Keywords:** infection, metagenomic next-generation sequencing, lung transplant recipients, pathogen, conventional detection methods

## Abstract

**Background:** Accurate identification of pathogens is essential for the diagnosis and control of infections. We aimed to compare the diagnostic performance of metagenomic next-generation sequencing (mNGS) and conventional detection methods (CDM) in lung transplant recipients (LTRs).

**Methods:** We retrospectively analyzed 107 LTRs with suspected infection of pulmonary, blood, central nervous system or chest wall between March 2018 and November 2020. Bronchoalveolar lavage fluid and other body fluids were subject to pathogen detection by both mNGS and CDM.

**Results:** Of the 163 specimens, 84 (51.5%) tested positive for both mNGS and culture, 19 (11.7%) of which were completely consistent, 44 (27.0%) were partially congruent, and 21 (12.9%) were discordant (kappa = .215; *p* = .001). Compared with CDM, mNGS detected a higher diversity of pathogens. Moreover, the turn-around time was significantly shorter for mNGS compared with culture (2.7 ± .4 vs. 5.5 ± 1.6 days, *p* < .001). As an auxiliary method, treatment strategies were adjusted according to mNGS findings in 31 cases (29.0%), including eight patients with non-infectious diseases, who were finally cured.

**Conclusion:** mNGS can identify pathogens with a shorter turn-around time and therefore provide a more accurate and timely diagnostic information to ascertaining pulmonary infections. mNGS might have a role in differentiating infectious from non-infectious lung diseases in LTRs.

## Graphical Abstract

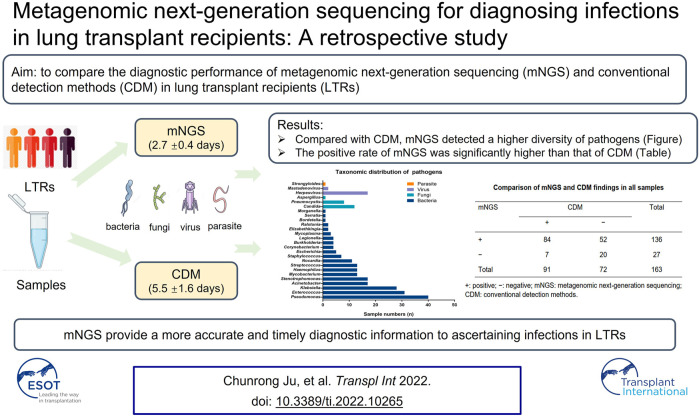

## Introduction

Infection is the main cause of death in lung transplant recipients (LTRs), especially at the early postoperative stages (1, 2). Compared with other solid organ transplant (SOT) recipients, LTRs are at significantly higher risk of acquiring infections because the lungs are constantly exposed to the atmospheric environment. This increased risk is further aggravated by the maintenance treatment with high-dose immunosuppressants, the impaired cough reflex, and the decreased mucociliary clearance especially at the early stage after lung transplantation (3, 4). The timely and accurate initiation of anti-infective treatment is vital to the clinical outcomes, which depends on the rapid and accurate pathogen identification. In real-world clinical practice, conventional detection methods (CDM) have a lower sensitivity and a relatively long turn-around time for detecting opportunistic pathogens such as *Pneumocystis jirovecii*, mycobacteria, *Nocardia* spp., fungi, and other atypical pathogens (5–7). Moreover, it is difficult to distinguish non-infectious diseases from infections because the clinical manifestations and radiologic characteristics are non-specific in LTRs (8–10). Therefore, accurate diagnosis of infection based on the exact identification of the pathogens are crucial to inform the decisions of therapeutic interventions.

Metagenomic next-generation sequencing (mNGS) is an emerging culture-independent assay that facilitates rapid and sensitive detection of various pathogens (5, 11). mNGS has recently been adopted for detecting pathogens in the respiratory, neurologic, urinary, pediatric, cardiovascular and orthopedic diseases (12–17). However, data among the LTRs have been scarce. The only existing study regarding mNGS mainly focused on the identification of viral species and explored the usefulness in LTRs with a previously undetectable source of infection (18). The value of mNGS for detecting other pathogens in LTRs has not been well elucidated. However, given the complexity of pathogens and the difficulty in differentiating the clinical diagnosis in LTRs, a thorough evaluation with mNGS is urgently needed.

In this study, we retrospectively analyzed the diagnostic performance of mNGS in diagnosing infectious diseases through the comparison with CDM. Our findings might help explore the role of mNGS in differentiating infectious from non-infectious pulmonary complication in LTRs.

## Materials and Methods

### Study Population

In this retrospective study, LTRs hospitalized in the First Affiliated Hospital of Guangzhou Medical University between March 2018 and November 2020 underwent screening. Inclusion criteria consisted of the following: 1) Aged 18 years or greater; 2) LTRs with new-onset pulmonary complication; and 3) BALF sample was available for pathogen detection by both mNGS and CDM. Patients with the undetermined diagnoses were excluded from our study.

Data of the LTRs that were collected retrospectively consisted of the demographics, primary underlying diseases before lung transplantation, the type of surgery (unilateral, bilateral, or heart-lung transplantation), clinical symptoms, signs, chest imaging findings, time from transplantation to sampling, laboratory routine tests, biochemical tests, treatment schemes (immunosuppressive and antimicrobial regimens) and clinical outcomes. All lungs were derived from the deceased cardiovascular or brain donors.

This study was carried out in accordance with the principles of the Declaration of Helsinki and was approved by the Ethics Review Committee of the First Affiliated Hospital of Guangzhou Medical University (No. 128, 2020). Patient approval and informed consent were waived because of the retrospective review of patient’s records.

### Criteria of Defining Pulmonary Infections

Pulmonary infection was diagnosed comprehensively according to the overall condition of the LTRs, which included the clinical manifestations (including symptoms), thoracic imaging, and laboratory findings, etc. We mainly took into account the thoracic imaging findings for diagnosing pulmonary infection. A new patchy or progressive infiltrate, consolidation, or ground-glass opacity should be shown on chest X-ray or computed tomography (CT). Meanwhile, patients would have to satisfy at least one of the following five items: 1) New-onset cough or expectoration, or aggravation of the existing respiratory tract symptoms with or without purulent sputum production, chest discomfort, dyspnea, or hemoptysis; 2) Fever; 3) Pulmonary consolidation and/or moist rales; 4) Peripheral blood white blood cell count >10 × 10^9^/L or <4 × 10^9^/L; 5) An evidence of pathogen infection. The differential diagnosis of infections and non-infectious diseases was established by combining the comprehensive clinical information and a review of the therapeutic outcomes.

### Sample Collection Schemes

Bronchoalveolar lavage fluid (BALF) samples were collected from patients with a new-onset pulmonary complication who were suspected as having infectious disease based on the overall clinical conditions. In addition, blood samples and cerebrospinal fluid (CSF) were collected from the patients who were suspected as having infection of the blood stream and the central nervous system, respectively. The exudate from the chest wall soft tissue mass was collected from patients suspected as having chest infections. The lung lobes with the most prominent lesions according to chest CT were selected for performing lavage with fiberoptic bronchoscopy according to the standardized operating procedures. 50–60 ml normal saline was instilled into the affected bronchial segment, with the target recovery rate of 40%–60%. Samples were immediately stored in sterilized containers and subjected to pathogen detection with CDM and mNGS.

The CDM included a minimal bundle of the bacterial and fungal smear and culture with the Grocott’s methenamine staining and acid-fast staining, real-time polymerase chain reaction (PCR) for cytomegaloviruses (CMV), Epstein-Barr virus (EBV), and *Mycobacterium tuberculosis* (TB)*,* serum antibody assays (with indirect immunofluorescence assay) for respiratory syncytial virus, influenza A/B virus, parainfluenza virus, adenovirus, *Legionella pneumophila*, *Mycoplasma pneumoniae*, and *Chlamydia pneumoniae*. In addition, galactomannan (GM) antigen and (1/3)-β-D-glucan (BDG) assays were adopted for detecting fungi. GeneXpert MTB/RIF, enzyme-linked immunospot assay (T-SPOT) and tuberculin skin test were performed among patients highly suspected as having TB. Meanwhile, an aliquot was stored at 4°C before immediately (within 4 h) being transferred to a designated central laboratory for performing mNGS. Trans-bronchoscopic lung biopsy (TBLB) was also performed among patients who could tolerate the procedure when non-infectious lung diseases (e.g., allograft rejection) were suspected.

### Sample Processing and DNA Extraction

The clinical samples mainly included BALF, peripheral blood, CSF, and exudate from the chest wall soft tissue mass. To prepare for the BALF samples, a 600 µl aliquot was aspired into a sterile container for breaking the cellular wall (esp. fungi), and another aliquot of 300 µl was subject to DNA extraction by using a TIANamp Micro DNA Kit (DP316; Tiangen Biotech, Beijing, China), according to the manufacturer’s instructions. For processing other samples such as the blood, CSF, and exudate from the chest wall soft tissue mass, 300–600 µl of samples was adopted.

The extracted DNA was subject to the comparison with the sequences in the genomic libraries through transposase indexing of each sample. After purification, amplification, and re-purification of the library, the fragment sizes and library concentrations were assessed by using Qsep1 (BiOptic, Hubei, China) and Qubit (Thermo Fisher Scientific, Waltham, MA, United States) devices, respectively. DNA nanoballs were prepared by using single-stranded DNA. Finally, each DNA nanoball was loaded into a single lane for sequencing. The sequencing was performed on the Illumina NextSeq 550Dx platform (Illumina, San Diego, CA, United States).

### Metagenomic Next-Generation Sequencing Data Analysis

Quality control was performed on the raw sequencing data by using the BWA platform (http://bio-bwa.sourceforge.net/). Low-quality reads and reads shorter than 35 bp were removed. The remaining reads were further filtered by using a human host DNA subtraction database. The sequences were then annotated by using a dedicated pathogen database after removing the low-complexity reads, and subsequently classified according to their taxonomic groups, such as viruses, bacteria, fungi, parasites, and other pathogens. The non-human sequence reads from each sample were deposited at the NCBI BioProject database (https://www.ncbi.nlm.nih.gov/bioproject) under the accession number PRJNA737316.

### Criteria for Defining Positive Findings of Metagenomic Next-Generation Sequencing

For mNGS assay, microorganism detection (bacteria, viruses and fungi) was considered positive if satisfying any of the following thresholds: 1) The relative abundance of bacteria (excluding *Mycobacterium tuberculosis* complex) and fungi was greater than 30% at the genera level; 2) Virus detection was considered when the stringent map read number (SMRN) was 3 or greater. 3) For *Mycobacterium tuberculosis* complex, at least one read should be aligned to the reference genome at species or the genera level (19) due to the technical challenges of DNA extraction and the low probability of contamination (20). However, positive mNGS finding did not invariably indicate the presence of causative pathogen, which required immediate treatment in clinical settings. It would be the clinician’s responsibility to determine the treatment strategy through comprehensive clinical assessments.

Microorganisms detected with mNGS were categorized into colonized microorganism, putative pathogen, and pathogenic microorganism. Torque teno virus, parvovirus, *Ureaplasma*, *Staphylococcus epidermidis*, intestinal colonized flora and anaerobic bacteria were deemed colonized microorganism should the patients remained clinically stable. Putative pathogens and pathogenic microorganisms were ascertained by two specialist clinicians according to the comprehensive assessments which consisted of the number of reads for mNGS, the clinical presentations, radiologic manifestations, conventional detection findings, and the clinical epidemiology. The putative pathogens or pathogenic microorganisms could be ascertained if consensus was achieved by the two clinicians. A third senior clinician and a fourth clinical microbiologist were further involved in the discussion in case of a major disagreement between the first two clinicians.

### Pathogens Identified by Conventional Detection Methods

Culture positive was considered if the microbial (bacterial and fungal) load exceeded 10^4^ CFU/ml. Positive BALF smear was defined as a Gram-positive and/or -negative bacterium or fungal spore/hyphae being detected by microscopic investigation. For fungi, both the positive results for BGD and GM antigen in the serum and the positive results for GM antigen in BALF were applied as the adjunct diagnostic criteria, except that pneumocystis was confirmed by PCR assay for the BALF samples. The targeted viruses, such as CMV or EBV, were detected with PCR assays of the BALF samples. The diagnosis of Mycobacterium infection was based on sputum smear for acid-fast bacilli, and the definitive diagnosis of TB or non-tuberculosis mycobacterium (NTM) was based on both culture and PCR, respectively. Moreover, the diagnosis of pulmonary TB was established according to the TB-related clinical symptoms, along with CT imaging findings and the results of the TB-spot and/or GeneXpert MTB/RIF.

### Statistical Analyses

Continuous variables were expressed as means ± standard deviation or median (IQR), and categorical variables as count (percentage). Paired McNemar chi-square tests and Cohens’ kappa were used to compare the difference and the concordance of mNGS with that of CDM. Statistical significance was defined at *p* < .05. Statistical analyses and plots were processed by using SPSS statistical software (IBM SPSS Statistics for Windows, Version 21.0. Armonk, NY, United States) and GraphPad Prism software (GraphPad Prism version 6.0.0 for Windows, GraphPad Software, San Diego, CA, United States).

## Results

### General Information of Study Participants

After screening for 266 LTRs, 107 eligible patients were included in our final analysis. The reasons for exclusion consisted of the following: mNGS not available for pathogen detection (*n* = 138) and unclear final diagnoses (*n* = 21). There were 90 males, and the mean age was 56.1 years. The mean body-mass index was 20.2 kg/m^2^. Of all LTRs, 60 underwent unilateral transplantation, 41 bilateral transplantation, and six combined heart-lung transplantation. The most common primary disease was interstitial lung disease (43.0%), followed by chronic obstructive pulmonary disease (33.6%). All LTRs received standard triple immunosuppressive regimens consisting of calcineurin inhibitors (tacrolimus/cyclosporin A), mycophenolate mofetil, and prednisolone. Table 1 demonstrates the characteristics of the LTRs.

**TABLE 1:** Patient and sample characteristics.

Characteristics	Value
Lung transplant recipients (*n* = 107)	
Age (years), mean ± SD	56.1 ± 13.3
Sex (male, %)	90 (84.1%)
BMI (kg/m^2^), mean ± SD	20.2 ± 3.6
Primary indications for lung transplantation, *n* (%)	
COPD	36 (33.6%)
Interstitial lung disease	46 (43.0%)
Bronchiectasis	10 (9.4%)
Pneumosilicosis	4 (3.7%)
Eisenmenger syndrome	4 (3.7%)
Pulmonary arterial hypertension	2 (1.9%)
BOS	2 (1.9%)
PLAM	1 (0.9%)
Re-transplantation	2 (1.9%)
Type of lung transplantation	
Unilateral lung transplantation	60 (56.1%)
Bilateral lung transplantation	41 (38.3%)
Heart−lung transplantation	6 (5.6%)
Total number of samples (*n* = 163)	
Sample type, *n* (%)	
BALF	159 (97.5%)
Blood	2 (1.2%)
CSF	1 (0.6%)
Exudate from the chest wall mass	1 (0.6%)
Time from transplant to sampling (days), median (IQR)	108 (18–419)
Clinical symptoms at sampling, *n* (%)	
Fever	21 (12.9%)
Cough/purulent sputum	134 (82.2%)
Dyspnea	74 (45.4%)
Chest tightness/pain	27 (16.6%)
Hemoptysis	6 (3.7%)
Headache	1 (0.6%)
Antimicrobial prophylaxis at sampling, *n* (%)	
^a^β-Lactams	134 (82.2%)
^b^Quinolones	21 (12.9%)
^c^Glycopeptides	52 (31.9%)
^d^Triazoles	123 (75.5%)
Ganciclovir	79 (48.5%)
^e^Other antibiotics	18 (11.0%)
None	9 (5.5%)

COPD, chronic obstructive pulmonary disease; CSF, cerebrospinal fluid; PLAM, pulmonary lymphangioleiomyomatosis; BOS, bronchiolitis obliterans syndrome.

a β-Lactam: including meropenem, imipenem, piperacillin and cefoperazone.

b Quinolones including moxifloxacin and levofloxacin.

c Glycopeptides including vancomycin and teicoplanin.

d Triazoles including voriconazole and posaconazole.

e Other antibiotics including trimethoprim‐sulfamethoxazole, minocycline and linezolid.

### Sample Types

BALF samples were collected from 106 LTRs (159 samples) at each episode of clinical exacerbation. Blood samples were collected from two patients who were suspected as having bloodstream infection, the exudate was sampled from one patient with a soft tissue mass on the chest wall, and CSF sample was collected from a patient suspected as having intracranial infection. Therefore, 163 specimens of different types were included in our analysis (Table 1).

### Spectrum of Pathogens Detected by Metagenomic Next-Generation Sequencing

**FIGURE 1:**
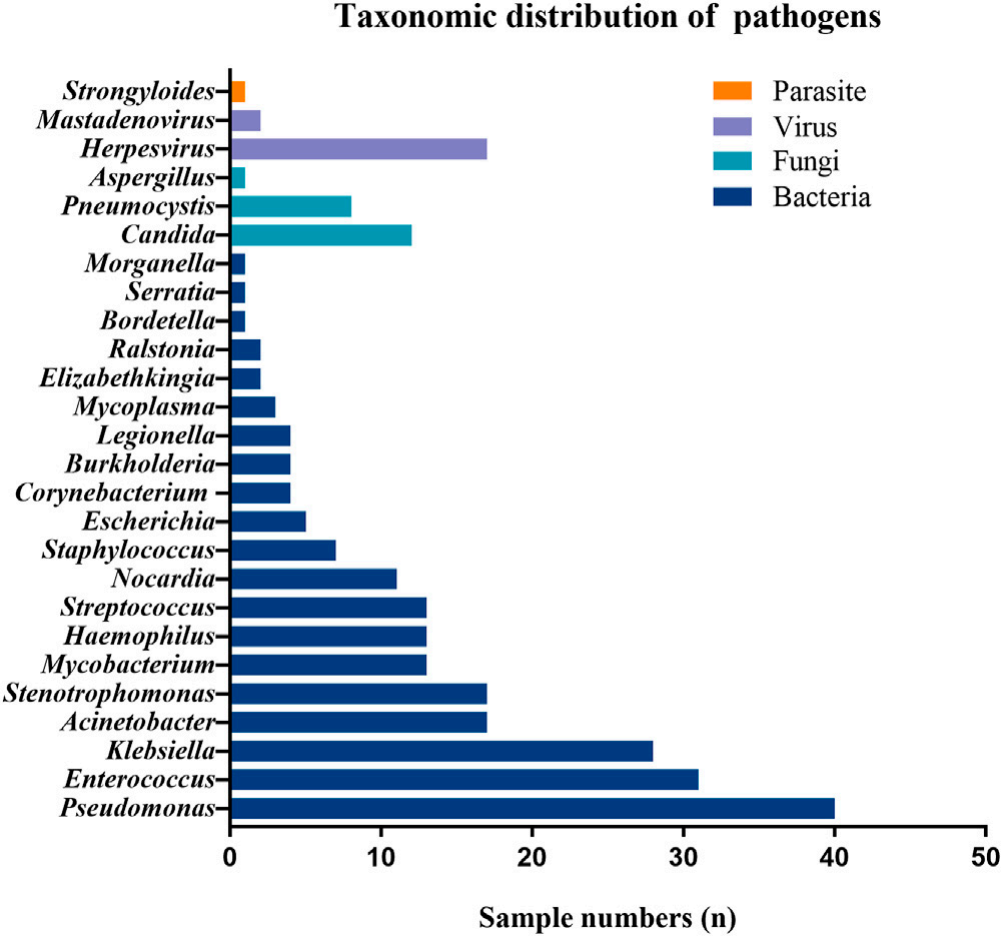
Taxonomic distribution of pathogens identified with mNGS in LTRs.

For the detection of pathogens in 163 specimens, 136 (83.4%) tested positive for mNGS with a significantly higher positive rate compared with CDM (83.4% vs. 55.8%, *p* = .027). Of these, 59 (36.2%) tested positive for a single pathogen and 77 (47.2%) for two or more pathogens. Herpesvirus was the most prevalent virus in BALF, whereas *Candida* was the most common fungi detected with mNGS. The three most common bacteria consisted of *Pseudomonas aeruginosa*, *Enterococcus* and *Klebsiella pneumoniae*. The detailed compositions of the putative pathogens detected with mNGS are demonstrated in Figure 1. Further details are shown in Supplementary Table S1.

Of the 163 specimens, 91 (55.8%) tested positive and 72 (44.2%) tested negative for CDM. Both CDM and mNGS tested positive among 84 samples (51.5%), and negative among 20 samples (12.3%). We also noted inconsistent findings among the two methods [negative CDM but positive mNGS findings in 52 (31.9%) samples, and positive CDM but negative mNGS findings in seven (4.3%) samples] (Table 2). The concordance of findings was moderate between mNGS and CDM findings (Cohen’s Kappa = .215; *p* = .001). The positive rate of mNGS was significantly higher than that of CDM (McNemar test *p* < .001; Table 2).

**TABLE 2:** Comparison of mNGS and CDM findings in all samples.

mNGS	CDM		Total
	+	−	
+	84	52	136
−	7	20	27
Total	91	72	163

+, positive; −, negative; mNGS, metagenomic next-generation sequencing; CDM, conventional detection methods.

**FIGURE 2:**
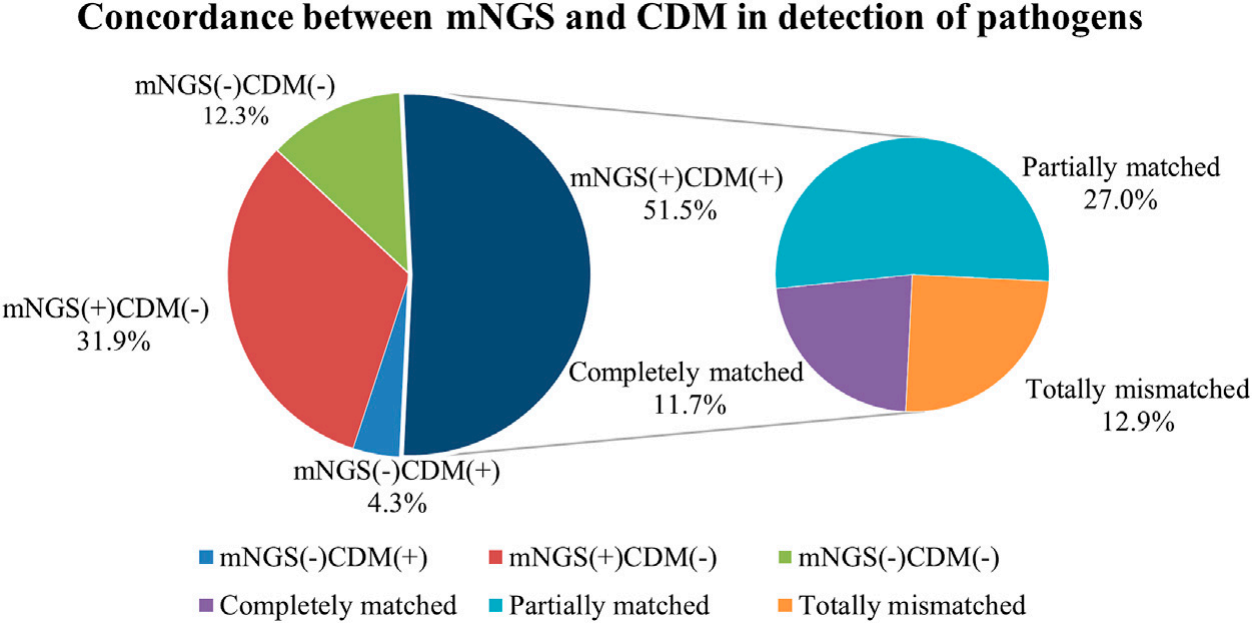
Pathogen detection congruence of mNGS and CDM.

The pathogens identified by CDM and mNGS were completely matched in 19 samples (11.7%). Of these, the three most common bacteria were *Pseudomonas aeruginosa* (*n* = 7), *Acinetobacter baumannii* (*n* = 5) and *Klebsiella pneumoniae* (*n* = 4). CDM findings were partially concordant with those of mNGS in 44 samples (27.0%). For instance, mNGS has revealed other pathogens (i.e., *Acinetobacter baumannii*) aside from the pathogens that were identified with culture alone (i.e. *Pseudomonas aeruginosa*). However, inconsistent findings were identified between mNGS and CDM in 21 samples (12.9%; Figure 2). In addition, mNGS was associated with a significantly shorter turn-around time as compared with CDM (2.7 ± .4 vs. 5.5 ± 1.6 days, *p* < .001).

Of the two blood samples, one was considered positive according to both mNGS and CDM which were consistently positive. These results were consistent with the clinical manifestations. For the other blood sample, the detection yielded inconsistent findings, with positive mNGS findings and negative blood culture findings. The pathogens detected by mNGS were *Klebsiella pneumoniae* and *Nocardia*, and the patient presented with the clinical manifestations of severe infection and sepsis. The pleural exudate sample tested negative for both mNGS and CDM. The single CSF sample tested positive for *Nocardia* with mNGS but not CDM (which did not reveal any pathogen). This occurred in a single patient who suffered repetitively from fever and headache for more than 1 month during which the pathogen had not been detected, with the clinical conditions worsening despite the use of broad-spectrum antibiotics.

### Treatment Adjustments According to the Positive Metagenomic Next-Generation Sequencing Findings

The treatment strategies were amended among 23 patients (21.5%) at an early stage based on the mNGS findings. Seven patients were diagnosed as having *Pneumocystis jirovecii* pneumonia, seven patients as having mycobacterial disease (including five patients with NTM pulmonary disease and two patients with pulmonary TB), four patients as having pulmonary nocardiosis, one patient as having legionellosis, one patient as having *Strongyloidiasis stercoralis* pneumonia, and one patient as having invasive pulmonary aspergillosis.

**FIGURE 3:**
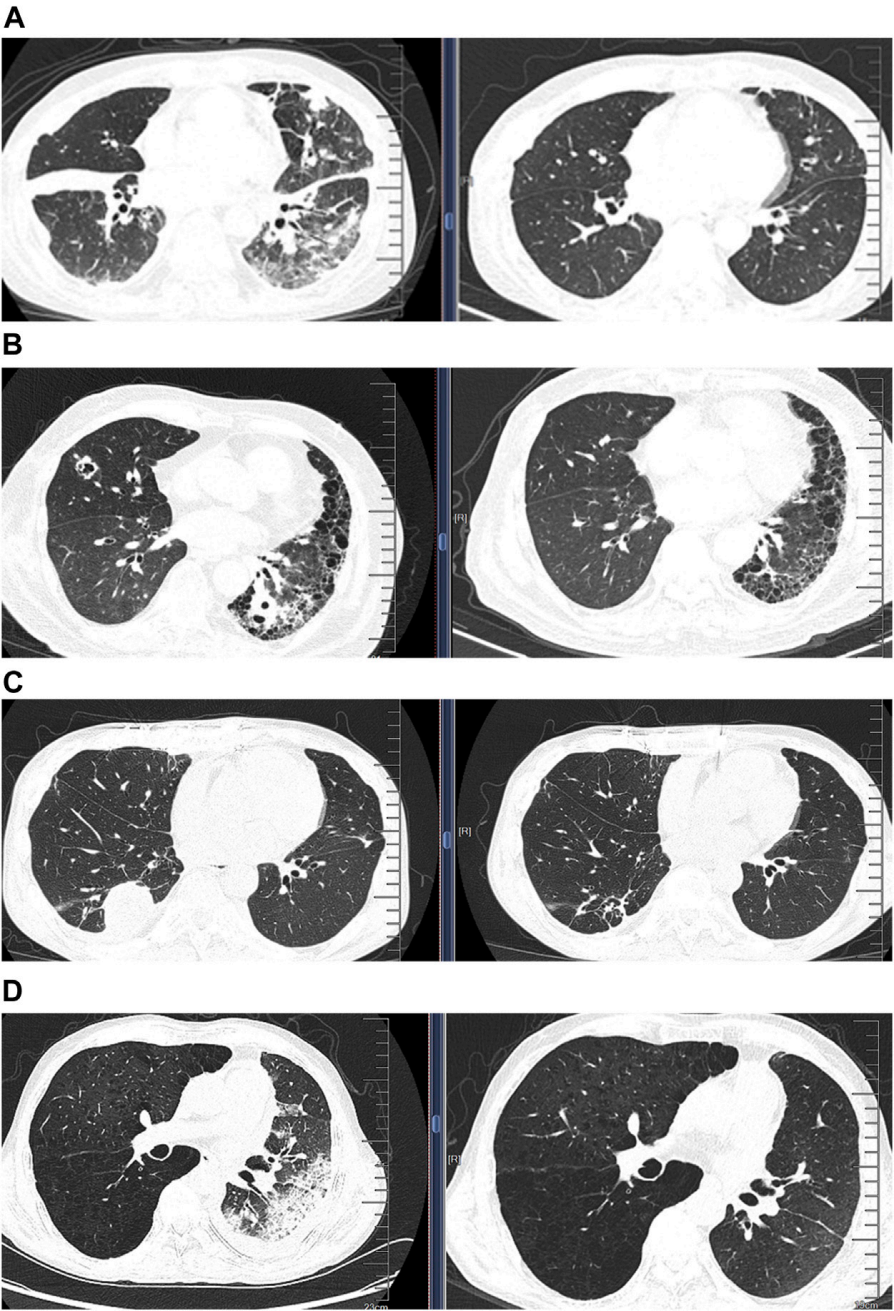
Comparison of chest computed tomographic (CT) images before and after treatment in four patients whose treatment regimens were switched thoroughly according to the mNGS findings. **(A)** CT images from a patient diagnosed as having *Pneumocystis jirovecii* pneumonia according to mNGS; CT images showing significant improvement of infiltration after treatment (right) compared with that before treatment (left); **(B)** CT images of disseminated nocardiosis before and after treatment; **(C)** CT images of NTM pulmonary disease before and after treatment; **(D)** CT images of acute rejection before and after treatment.

Moreover, one patient was treated immediately according to the blood mNGS findings who had been confirmed to have suffered from *Klebsiella pneumoniae* bloodstream infection according to the clinical manifestations and the delayed culture findings. For the patient whose CSF tested positive for *Nocardia* by mNGS, cotrimoxazole and linezolid were administered immediately, after which the clinical condition improved significantly within 1 week until clinical cure. Figures 3A–C shows the comparison of chest CT images before and after treatment in the three LTRs whose treatment strategy switched from the initial anti-infectious regimens into a different anti-infectious regimen according to the mNGS findings (Supplementary Table S2).

### Negative Metagenomic Next-Generation Sequencing Fingdings as an Auxiliary Diagnosis of Non-Infectious Pulmonary Disease

We finally analyzed the negative mNGS findings as an auxiliary diagnosis of non-infectious diseases. Among the eight cases (7.5%) who yielded negative mNGS findings, one was eventually diagnosed as having pulmonary mucinous adenocarcinoma based on TBLB histopathology. Six patients were suspected as having acute rejection according to the comprehensive assessment of clinical characteristics; however, biopsy was not possible due to the poor clinical conditions. Because of the absence of pathological evidence, the patients were deemed to have acute rejection according to the negative mNGS findings along with the clinical manifestations. Therefore, the treatment strategies switched from antibiotics to an escalation of the dose of immunosuppressants. This led to the progressively improved clinical conditions and significantly diminished pulmonary infiltration, which collectively indicated resolved acute rejection (Figure 3D; Supplementary Table S2). The remaining one patient who had a soft tissue mass on the chest wall had initially been prescribed with antibiotics which was subsequently withheld because of the negative mNGS findings. The soft tissue mass was diagnosed to be local lymphatic fistula, and the exudate finally dissipated.

## Discussion

We have for the first time delineated the strengths of mNGS for ascertaining the infection status and pathogen identification in LTRs. We have also explored the diagnostic performance of mNGS as an auxiliary diagnostic approach of non-infectious complications, revealing how the treatment strategies could be amended by taking into account the findings from mNGS.

In this study, mNGS was employed to identify the pathogens in various body fluid samples, revealing a significantly higher positive rate and diversity compared with CDM. Our findings were in line with those of other recent mNGS studies (19–22), suggesting that mNGS could result in a higher positive rate and a greater accuracy of diagnosing pulmonary infection in LTRs. There may be two explanations for these outcomes: 1) mNGS can detect a wide range of pathogenic and non-pathogenic microorganisms which reside within the lungs; 2) mNGS might be capable of detecting dead pathogens whereas culture could only identify live microorganisms. Thus, whether the microorganisms detected by mNGS are causative or colonized pathogens should be determined by clinicians based on the comprehensive assessment of the clinical information.

Our results supported the assumption that mNGS has considerable advantages over CDM (6). While some shortcomings of mNGS such as higher cost need to be resolved before the extensive application as a reliable routine diagnostic method in LTRs. However, mNGS is characterized by the rapid turn-around time which takes from less than 3 days to, until recently, within 24 h only. Accurate administration of antibiotics is important for improving the prognosis among LRTs with infections. However, this depends heavily on the early identification of pathogens (23). Our results were consistent with those of the recent studies which showed that mNGS could be used for diagnosing clinical infectious diseases with the advantages of a high throughput, rapid turn-around, and high sensitivity (19, 21, 22). Taken together, mNGS confers considerable advantages over CDM for diagnosing pulmonary infections in LTRs.

In addition, compared with CDM, mNGS yielded a significantly higher sensitivity for the sample types other than respiratory specimens, such as blood and CSF (13, 24). For bloodstream infections, mNGS was less affected by the previously administered antibiotics compared with culture (24–26), which might help interpret why mNGS also yielded a higher sensitivity. In fact, most LTRs were treated with antibiotics at the time of specimen collection.

For diagnosing pathogen which was responsible for pulmonary infection, mNGS assays showed that the most prevalent pathogens mainly consisted of bacteria, particularly in LTRs at the early post-lung transplantation stages, which was in line with the results of several studies (27–29). The three most prevalent bacteria were *Pseudomonas aeruginosa*, *Enterococcus* and *Klebsiella pneumoniae*. Although mNGS can help detect clinically common multi-drug resistant bacteria with a higher sensitivity compared with conventional culture, the CDM could determine antibiotic sensitivity which cannot be achieved by mNGS. Therefore, the selection of antibiotics in our study was mainly based on culture, and our results concurred with the opinion that culture methods might be more informative than mNGS for detecting bacterial drug-resistance (30).

*Pneumocystis jirovecii* is one of the most common opportunistic pathogens in LTRs. However, the low rate of confirmed diagnoses as revealed with CDM could readily result in a high mortality rate. In our study, seven LTRs were diagnosed as having pneumocystis jirovecii pneumonia based on mNGS results, which were verified by PCR subsequently. The LTRs were cured after a timely adjustment of treatment with sulfamethoxazole-trimethoprim. Our results were in line with previous studies, suggesting that mNGS would be a promising method for rapid and accurate detection of *Pneumocystis jirovecii* (31, 32). Our findings supported the conclusions of the previous studies which posited that patients would benefit from mNGS assay due to the high sensitivity of pathogen detection (33, 34).

Due to the non-specific clinical manifestations and the low positive rates, nocardiosis cannot be readily diagnosed or is prone to be misdiagnosed in clinical settings (35, 36). In our study, a patient with cerebral nocardiosis suffered from refractory fever and headaches for more than 1 month, mNGS finally unraveled the culprit pathogen within the CSF. Furthermore, another patient with disseminated nocardiosis, the diagnosis was entirely based on mNGS findings. In addition, other pathogens such as *Legionella pneumoniae*, *Mycoplasma pneumoniae*, and *Strongyloidiasis stercoralis* were detected in BALF by mNGS but not CDM. Our findings suggested a considerable clinical value of mNGS for diagnosing the infections with rare pathogens and the atypical pathogens which were associated with the low detection rates according to the CDM. Other studies have also shown a higher positive rate of detection for certain fungal species and some rare pathogens with mNGS (25, 37–39).

It was worth noting that there were seven LTRs who showed lung infiltration in the chest CT, but pathogen detection in BALF using mNGS was negative. It was challenging to obtain lung tissue biopsy samples, and the six patients were diagnosed as having probable acute rejection based on their overall clinical manifestations. After initiating the immunosuppressive therapy, the pulmonary infiltration was well absorbed, and the clinical condition improved considerably. In another patient, the negative mNGS finding from BALF samples have informed physicians to perform invasive biopsy although the patient might not tolerate the procedures. The final diagnosis was confirmed to be lung adenocarcinoma according to the pathology findings. Therefore, our study results suggested that negative mNGS results might also be useful for the differential diagnosis of infectious and non-infectious pulmonary complications after lung transplantation.

### Limitations

First, in this retrospective study, most patients had already received antibiotic treatment prior to collecting the samples which might have resulted in a decreased positivity rate compared with CDM. Second, samples were collected only at the initial stage of the disease for comparison with CDM. Due to the high cost of mNGS, we did not perform mNGS to test RNA virus and no longitudinal comparison was performed after the condition had improved. Finally, ascertaining the putative pathogen of infection should be made in conjunction with the clinical manifestations, the findings of both mNGS and CDM, while the interpretation of mNGS findings depends on the clinician’s expertise, therefore some bias may still remain.

## Conclusion

Compared with CDM, mNGS is associated with a higher diagnostic yield of identifying infection and could help differentiate infectious from non-infectious diseases in LTRs. Because of the advantages such as the short turn-around time and the high sensitivity, mNGS might be further pursued as a routine approach for the management of LTRs.

## Capsule Sentence Summary

Infection is the predominant cause of death in lung transplant recipients, timely and accurate anti-infection schemes are vital to ensure the best possible treatment outcomes. However, it is difficult to detect some pathogens using conventional detection methods in clinical practice for various reasons, and conventional culture suffers from the limitations such as being time-consuming. Thus, the diagnosis of lung infection and identification of pathogens is crucial for determining the treatment options in this population. As far as we know, this is first investigation on the clinical application of metagenomic next-generation sequencing (mNGS) in lung transplant recipients. In this study, we collected 159 bronchoalveolar lavage fluid (BALF) and four samples of other body fluid in lung transplant recipients. We found that mNGS detection sensitivity of plumonary infections in lung transplant recipients was significantly higher than that of conventional detection methods. In particular, mNGS revealed the infection of some pathogens that were difficult to detect using conventional detection methods, including *Pneumocystis jirovecii*, mycobacteria, and *Nocardia*. mNGS offers not only a substantially higher diagnostic sensitivity with a more rapid diagnosis of infectious diseases, but can also help differentiate infectious from non-infectious lung diseases in lung transplant recipients.

## Data Availability

The datasets presented in this study can be found in online repositories. The names of the repository/repositories and accession number(s) can be found below: https://www.ncbi.nlm.nih.gov/, PRJNA737316.

## Abbreviations

BALF, bronchoalveolar lavage fluid; BDG, (1/3)-β-D-glucan; BOS, bronchiolitis obliterans syndrome; CDM, conventional detection methods; CMV, cytomegaloviruses; COPD, chronic obstructive pulmonary disease; CSF, cerebrospinal fluid; CT, computed tomography; EBV, Epstein-Barr virus; GM, galactomannan; LAM, Lymphangioleiomyomatosis; LTRs, lung transplant recipients; mNGS, metagenomic next-generation sequencing; NTM, non-tuberculosis mycobacterium; PCR, polymerase chain reaction; SOT, solid organ transplant; TBLB, trans-bronchoscopic lung biopsy.
