# Supplementary material for: Metagenomic Next-Generation Sequencing for Diagnosing Infections in Lung Transplant Recipients: A Retrospective Study
Source: Transpl Int. 2022 Feb 10;35:10265. doi: 10.3389/ti.2022.10265 (PMC8866178; doi:10.3389/ti.2022.10265)
Supplement: Supplementary file 1 [file Table1.docx]

**Additional file**

**Supplementary Table S1 Putative pathogens detected by mNGS**

|  | **Genus (NO)** | **Species (NO)** |
| --- | --- | --- |
| Parasite | *Strongyloides* (1) | *Strongyloides stercoralis* (1) |
| Virus | *Mastadenovirus* (2) | *adenovirus* (2) |
|  | *Herpesvirus* (17) | *Cytomegalovirus* (11); *Epstein-barr virus*(2) ; *Human herpesvirus* 7 (4) |
| Fungi | *Aspergillus* (1) | *Aspergillus fumigatus* (1) |
|  | *Pneumocystis* (8) | *Pneumocystis jirovecii* (8) |
|  | *Candida* (12) | *Candida albicans* (9)；*Candida tropicalis* (1)；*Candida parapsilosis* (1)；*Candida dubliniensis*（1） |
| Bacteria | *Morganella* (1) | *Morganella morganii* (1) |
|  | *Serratia* (1) | *Serratia marcescens* (1) |
|  | *Bordetella* (1) | *bordetella parapertussis* (1) |
|  | *Ralstonia* (2) | *Ralstonia mannitolilytica* (1); *Ralstonia pickettii* (1) |
|  | *Elizabethkingia* (2) | *Elizabethkingia anopheles* (2); |
|  | *Mycoplasma* (3) | *Mycoplasma hominis* (3); |
|  | *Legionella* (4) | *Legionella pneumophila* (3); *Legionella anise* (1) |
|  | *Burkholderia* (4) | *Burkholderia multivorans* (1)；*Burkholderia cepacian* (3) |
|  | *Corynebacterium* (4) | *Corynebacterium resistens* (1)；*Corynebacterium striatum* (3) |
|  | *Escherichia* (5) | *Escherichia coli* (5) |
|  | *Staphylococcus* (7) | *taphylococcus aureus* (7) |
|  | *Nocardia* (11) | *Nocardia farcinica* (10)；*Nocardia serosa* (1) |
|  | *Streptococcus* (13) | *Streptococcus pneumoniae* (13) |
|  | *Haemophilus* (13) | *Haemophilus parahaemolyticus* (11)；*Haemophilus influenzae* (1)；*Haemophilus influenzae* (1) |
|  | *Mycobacterium* (13) | *Mycobacterium avium* (2)；*Mycobacterium abscessus* (6)；*Mycobacterium gordonae* (1)；*Mycobacterium tuberculosis* (4) |
|  | *Stenotrophomonas* (17) | *Stenotrophomonas maltophilia* (17) |
|  | *Acinetobacter* (17) | *Acinetobacter baumannii* (17)； |
|  | *Klebsiella* (28) | *Klebsiella pneumoniae* (28) |
|  | *Enterococcus* (31) | *Enterococcus faecium* (18)；*Enterococcus avium* (4)；*Enterococcus faecalis* (8)；*Enterococcus caseosa* (1) |
|  | *Pseudomonas* (40) | *Pseudomonas aeruginosa* (40) |

mNGS: metagenomic next-generation sequencing
