# Supplementary material for: Metagenomic Next-Generation Sequencing for Diagnosing Infections in Lung Transplant Recipients: A Retrospective Study
Source: Transpl Int. 2022 Feb 10;35:10265. doi: 10.3389/ti.2022.10265 (PMC8866178; doi:10.3389/ti.2022.10265)
Supplement: Supplementary file 2 [file Table2.docx]

**Additional file**

**Supplementary Table S2 The mNGS results led to change in treatment strategies**

| **Patient no.** | **CDM results** | **mNGS findings** | **Changes in treatment strategies** |
| --- | --- | --- | --- |
| 9 | *Klebsiella pneumoniae;* BDG: 365pg/ml; LDH: 442U/L; *P. jirovecii* DNA: positive. | *Pneumocystis jirovecii, Klebsiella pneumoniae* | Supplementation of sulfamethoxazole |
| 10 | BDG:＜10pg/ml; LDH:407U/L; *P. jirovecii* DNA: positive. | *Pneumocystis jirovecii* | Supplementation of Sulfamethoxazole |
| 13 | BDG:＞1000pg/ml; LDH:1108U/L; *P. jirovecii* DNA: positive. | *Pneumocystis jirovecii,* cytomegalovirus | Switched to Sulfamethoxazole, Caspofungin and Ganciclovir |
| 15 | *Acinetobacter baumanni*, *Pseudomonas aeruginosa* | *Nocardia farcinica* (bronchoalveolar lavage fluid + blood), *Acinetobacter baumanni*, *Pseudomonas aeruginosa*, cytomegalovirus | Switched Meropenem to Imipenem, added Linezolid, Ganciclovir |
| 21 | Negative | Negative | Supplementation of high does intravenous methylprednisolone for acute rejection |
| 23 | BDG:＜10pg/ml; LDH: 376.2U/L;  *P. jirovecii* DNA: positive. | *Pneumocystis jirovecii*, cytomegalovirus, Epstein-Barr virus | Switched to Sulfamethoxazole, Caspofungin and Ganciclovir |
| 27 | *Enterococcus faecium*; GeneXpert MTB/RIF (+) | *Mycobacterium tuberculosis complex* | Supplementation of anti-TB drugs (isoniazide, pyrazinamide, ethambutol) |
| 28 | Negative | Negative | Discontinuation of antibiotics, performed lung biopsy and diagnosed as having lung cancer |
| 29 | BDG:＜10pg/ml; LDH: 343.5U/L;  *P. jirovecii* DNA: positive. | *Pneumocystis jirovecii* | Supplementation of Sulfamethoxazole |
| 31 | Negative | *Nocardia farcinica* | Switched to imipenem and linezolid |
| 32 | Negative | Negative | Supplementation of high does intravenous methylprednisolone for acute rejection |
| 37 | *Pseudomonas aeruginosa* | *Mycobacterium abscessus, Pseudomonas aeruginosa, Klebsiella pneumoniae,* Adenoviridae | Supplementation of Linezolid and Clarithromycin |
| 40 | Negative | Negative | Switched to high-dose intravenous methylprednisolone for acute rejection |
| 45 | *Stenotrophomonas maltophilia* | *Mycobacterium chelonae, Stenotrophomonas maltophilia, Pseudomonas aeruginosa, Staphylococcus aureus* | Supplementation of Linezolid and Clarithromycin |
| 47 | Negative | *Klebsiella pneumoniae* (blood) | Switched from immunosuppressants (anti-rejection) to anti-infection (Ceftazidime avibatan) |
| 48 | BDG:＜10pg/ml; LDH: 408U/L;  *P. jirovecii* DNA: positive. | *Pneumocystis jirovecii* | Switched to Sulfamethoxazole and Caspofungin |
| 51 | GeneXpert MTB/RIF (+); acid-fast staining (++) | *Mycobacterium tuberculosis complex* | Supplementation of anti-TB drugs (isoniazide, pyrazinamide, ethambutol) |
| 52 | Negative | Negative | Supplementation of high does intravenous methylprednisolone for acute rejection |
| 56 | Negative | Negative | Supplementation of high does intravenous methylprednisolone for acute rejection |
| 60 | *Candida albicans* | *Strongyloides stercoralis, Candida albicans* | Supplementation of Ivermectin |
| 61 | *Enterobacter cloacae* | *Mycobacterium abscessus* | Supplementation of Linezolid and Clarithromycin |
| 74 | Negative | *Legionella pneumophila* | Switched from Piperacillin sodium tazobactam sodium to Azithromycin and Moxifloxacin |
| 75 | Negative | *Nocardia farcinica,* cytomegalovirus | Switched from Piperacillin sodium tazobactam sodium to Imipenem and Linezolid |
| 76 | *Stenotrophomonas maltophilia, Staphylococcus haemolyticus* | *Mycobacterium abscessus, Stenotrophomonas maltophilia,* | Supplementation of Linezolid and Clarithromycin |
| 85 | *Klebsiella pneumoniae* | *Mycobacterium abscessus, Klebsiella pneumoniae, Enterococcus faecium* | Supplementation of Linezolid and Clarithromycin |
| 86 | Negative | *Nocardia farcinica* | Supplementation of Sulfamethoxazole and Linezolid |
| 89 | Negative | Negative (chest wall mass exudate) | Discontinuatoin of antibiotics |
| 94 | BDG:126.5pg/ml; LDH: 162U/L;  *P. jirovecii* DNA: positive. | *Pneumocystis jirovecii, Pseudomonas aeruginosa* | Supplementation of Sulfamethoxazole and Caspofungin |
| 95 | *Stenotrophomonas maltophilia*; *Aspergillus fumigatus*; BALF GM(+) | *Aspergillus fumigatus, Stenotrophomonas maltophilia* | Supplementation of Voriconazole |
| 100 | Negative | Negative | Supplementation of high does intravenous methylprednisolone for acute rejection |
| 107 | Negative | *Nocardia* *farcinica* (cerebrospinal fluid) | Switched to Sulfamethoxazole and Linezolid |

Conventional detection methods (CDM) included bacterial and fungal smear and culture with the Grocott’s methenamine staining and acid-fast staining, real-time polymerase chain reaction (PCR) for cytomegalovirus, Epstein-Barr virus, and *Mycobacterium tuberculosis, Pneumocystis jirovecii* (*P. jirovecii*); serum antibody assays (with indirect immunofluorescence assay) for respiratory syncytial virus, influenza A/B virus, parainfluenza virus, adenovirus,*Legionella pneumophila*, *Mycoplasma pneumoniae*, and *Chlamydia pneumoniae*. Galactomannan (GM) antigen and (1/3)-β-D-glucan assays (BDG) were also adopted for detecting fungi. GeneXpert MTB/RIF, enzyme-linked immunospot assay (T-SPOT) and tuberculin skin test were only performed for patients highly suspected as having tuberculosis (TB).

Normal Range: lactate dehydrogenase (LDH):109~255U/L; BGD:＜100pg/ml.
